# Supplementary material for: An integrative, multi-scale, genome-wide model reveals the phenotypic landscape of Escherichia coli
Source: Mol Syst Biol. 2014 Jul 1;10(7):735. doi: 10.15252/msb.20145108 (PMC4299492; doi:10.15252/msb.20145108)
Supplement: Supplementary file 1 — Supplementary Figures S1-S24 [file msb0010-0735-sd1.docx]

**Supplementary Figure S1**: A novel gene expression (*Eco*MAC) and phenomics (*Eco*Phe) database of *E. coli* (A), which contains 2,198 microarrays divided into different categories depending on the perturbations included in each array (B).

**Suppl. Fig. 2**: Gene expression diversity observed in 31 different *E. coli* strains included in *Eco*MAC. Gene expression variability was measured as the relative error (A) or the *PCC* (B) between conditions with different strains (right panel) and the WT expression profile. Errors bars represent SD.

**Supplementary Figure S3**: Gene expression diversity observed in *E. coli* cells growing in 15 different media. Gene expression variability was measured as the relative error (A) or the *PCC* (B) between conditions with different media (right panel) and the WT expression profile. Errors bars represent SD.

**Supplementary Figure S4**: Gene expression diversity under all different environments, i.e., media (332 arrays), and genetic perturbations (718 arrays) annotated in *Eco*MAC measured as the *PCC* (A) or the relative error (B) between the perturbed conditions and the wild-type expression profile. *P_1_* and *P_2_* denote *P*-values of the Kolmogorov-Smirnov and Mann-Whitney tests, respectively, to compare gene expression diversity between environmental and genetic perturbations. (C, D) Gene expression diversity under different types of genetic modifications (KO, gene knockout; OE, over-expression; transcriptional rewiring of several TFs) identified in TFs, enzymes or the rest of genes. Errors bars represent SD.

**Supplementary Figure S5: (**A) ROCs for the 3 inference method consensus results using *Eco*MAC, the results from DREAM5 community inferred network, and the 3 same methods consensus network, using the DREAM5 dataset against RegulonDB v8.1 confirmed connections (566). (B) Precision-Recall curve corresponding to (A). (C) ROCs for the 3 inference method consensus results using *Eco*MAC, the results from DREAM5 community inferred network, and the 3 same methods consensus network, using the DREAM5 dataset against RegulonDB 8.1 strong connections (3,083). (D) Precision-Recall curve corresponding to (C). (E) ROCs for the 3 inference method consensus results using *Eco*MAC, the results from DREAM5 community inferred network, and the 3 same methods consensus network using the DREAM5 dataset against RegulonDB v6.8 DREAM5 golden standard. (F) Precision-Recall curve corresponding to (E). (F) Gene regulatory network for 500 top ranked inferred connections (*Eco*MAC), corresponding to 45% precision.

**Supplementary Figure S6**: Gene expression correlation between TFs and their targets in the experimental TRN (A and C; only 3,011 interactions where the *PCC* was statistically significant (*p* < 0.01)) and the experimental and inferred TRN (B and D; only 4,447 interactions where the *PCC* was statistically significant (*p* < 0.01)). Histograms of the *PCC* (A-B); blue and red bars/points represents the interactions of the *E. coli* TRN and random interactions respectively. Average and SD of the previous distributions (C and D). *P_1_* and *P_2_* denote *P*-values of the Kolmogorov-Smirnov and Mann-Whitney tests, respectively, to compare gene expression correlation between TFs and their targets with random interactions.

**Supplementary Figure S7**: Transcriptional mechanisms to sense environmental signals (A). Four different types of signal transduction mechanisms identified in *E. coli*: (*i*) one-component signal transduction system where a TF is negatively auto-regulated and this repression is reduced by the formation of an inactivated complex between the effector and the TF (B); (*ii*) one-component signal transduction system where a TF is negatively auto-regulated by the formation of an active complex between the effector and the TF (C); (*iii*) two-component signal transduction system (D); (*iv*) unknown mechanism (E).

**Supplementary Figure S8**: Examples of the transcriptional mechanism of two STSs: Type I (A) and II (B).

**Supplementary Figure S9**: Histogram of parameter ${\Delta n}_{E}^{max}$ for 151 STSs.

**Supplementary Figure S10**: Gene expression capacities. (A) Distribution of the expression profile of a given gene across the whole condition of *Eco*MAC. Red area represents a percentage of experimental conditions in which gene expression is not compatible with the gene expression capacities. (B) Number of conditions excluded for different values of the gene expression capacities. Error bars represent SD for all genes.

**Supplementary Figure S11**: Performance of the predicted transcriptional activators and repressors of *E. coli*. ROC-curve areas of the predicted activators or repressors (A-B) or the average of both areas (C-D) represent the predictive power for inferring those regulators by using linear regression (A and C) or multiple linear regression (B and D) for different sets of interactions defined by having a *PCC* higher than a given threshold.

**Supplementary Figure S12**: Histograms of the regulatory coefficients (A) and basal gene expression (B) of the TRN of *E. coli*. Number of interactions categorized as activations (C), repressions (D), and the ratio between repressions and activations (E) in the experimental TRN. Dashed lines represent the number of activations and repressions observed in RegulonDB and the ratio of repressions and activations consistent to the “Demand theory” (Savageau, 1998a; Savageau, 1998b). Blue and red bars represent the model with a topology defined by the experimental, or both experimental and inferred interactions, respectively. *P_1_* and *P_2_* denote *P*-values of the Kolmogorov-Smirnov and Mann-Whitney tests, respectively, to compare model parameters between the experimental, and experimental and inferred TRN.

**Supplementary Figure S13**: Sensitivity analysis of the EBA predictive power. Percentage of well-predicted arrays with respect to the first null model (Fig. 4A, black bars) to predict genetic (A) and environmental (B) perturbations by using the inferred parameters or random values in a range extended of the inferred parameters. Only experimentally validated interactions are included. *PCC* was computed between predicted and the experimental expression profiles evaluating all genes (global scores) or selecting only a specific set of genes (local scores). Note that gene expression profiles validated by using local* scores only considered well-predicted arrays if *PCC* was statistically significant (*p* < 0.05) additionally to the criteria defined in the Section 4.4.2.

**Supplementary Figure S14**: Predictive power of EBA by including only experimental (A-B), or both inferred and experimental (C-D) interactions. All three null models were considered. We compared two scoring functions to evaluate the predicted gene expression profiles: *PCC* (A and C) and relative error (B and D) computed between predicted and the experimental expression profiles evaluating all genes (global scores) or selecting only a specific set of genes (local scores). Note that gene expression profiles validated by using local* scores only considered well-predicted arrays if *PCC* was statistically significant (*p* < 0.05) additionally to the criteria defined in the Section 4.4.2.

**Supplementary Figure S15**: Dissecting the predictive power of EBA (including only experimental interactions) methodology under different categories of genetic perturbations: all genetic perturbations (A), gene knockouts (B), gene over-expression (C), and rewired networks (D). We used *PCC* to compute the predicted gene expression profiles by evaluating all genes (global scores) or selecting only a specific set of genes (local scores). Note that gene expression profiles validated by using local* scores only considered well-predicted arrays if *PCC* was statistically significant (*p* < 0.05) additionally to the criteria defined in the Section 4.4.2. Red lines dashed show the percentage average for each case (global, local, local with significantly better PCC).

**Supplementary Figure S16**: Topology analysis (A-B) and 5-fold cross-validation (C-D) of the predictive power of EBA (including only experimental interactions). For the topology analysis, we used *PCC* (green) and relative error (orange) to evaluate the distance between the predicted and experimental gene expression profiles evaluating all genes (global scores) or selecting only a specific set of genes (local scores). Note that gene expression profiles validated by using local* scores only considered well-predicted arrays if *PCC* was statistically significant (*p* < 0.05) additionally to the criteria defined in the Section 4.4.2.

**Supplementary Figure S17**: Metabolic benefit predicted under random environments. The environments are simulated by a core of nutrients based on those nutrients found in a minimal media with the addition of random carbon (A), nitrogen (B), amino acids (C), and metal (D) sources. (A-D) Predicted metabolic benefits as percentage of the sources added in the environments. 100% represents the addition of all influx carbon sources. Error bars are SD of metabolic benefits across 100 random environments. Carbon sources are the raw ingredients for growth, so increasing these would naturally allow higher growth rates. In the case of Nitrogen, the benefit does not increase linearly, because of the limitation in other carbon sources. Supplemental amino acid sources are the raw ingredients for the growth rate, so increasing these would naturally allow higher growth rates. Metals generally show zero growth rates until the higher percentages, because a large number of metals are required for survival.

**Supplementary Figure S18**: Metabolic benefit (or variation in biomass) predicted under single genetic perturbations (blue line), enzyme knockouts (A), and over-expression (B). For every gene perturbation, it is showed the percentage of environments that caused an intermediate growth rate between 0 and the wild-type growth rate (red line). Error bars denote the SD of metabolic benefit across 100 random environments.

**Supplementary Figure S19**: Metabolic benefit predicted under multiple genetic perturbations (blue line), TF knockouts (A), and over-expression (B). For every TF perturbation, it is showed the percentage of environments that caused an intermediate growth rate between 0 and the wild-type growth rate (red line). Error bars denote the SD of metabolic benefit across 100 random environments.

**Supplementary Figure S20**: Integration of the four sub-models (signal transduction, transcription, metabolism, and phenotype) of the *E. coli* integrative genome-scale model.

**Supplementary Figure S21**: Phenotype predictions for arrays in *Eco*Phe containing genetic and environmental perturbations by using the integrative genome-scale model of *E. coli* in which we use (A) the measured expression profile from *Eco*MAC; (B) the EBA model with only experimental interactions and (C) the EBA model with both experimental and inferred interactions. *PCC* is shown between the growth rates measured and predicted by the cost function, the metabolic benefit or the benefit – cost model (* *p* < 10^-2^, ** *p* < 10^-3^, *** *p* < 10^-10^). Note that the numbers below the panels show the size of the optimal subsets of arrays, as explained in section 6.2. Black bars represent the null-model to predict growth rates. (D) Scatter plot of the growth rates measured and predicted in (C).

**Supplementary Figure S22**: Phenotype predictions for arrays in *Eco*Phe to compare different categories of perturbations (low *vs* high growth rate measured; genetic *vs* environmental perturbations; gene knockouts *vs* rewired networks) by using the integrative genome-scale model of *E. coli* in which: (A) EBA was replaced by the expression profiles from *Eco*MAC; (B) EBA with experimental and inferred interactions, predicted gene expression profiles. Top panels show the *PCC* between the growth rates measured and predicted by the benefit – cost model, and bottom panels illustrate the measured and predicted growth rates for all arrays grouped in each category.

**Supplementary Figure S23**: (A) Histogram of number of genes that could be annotated in different GO terms. (B) Histogram of the number of GO terms with different sizes. (C-E) Set of genes proposed to be knockout by the implemented Greedy algorithm to maximize the number of GO terms to be altered subject to minimize the number of genes to include in the proposed set. We used three GO term rules to characterize as an altered biological process: (C) 1, 2, and 3 or more genes of the GO term are perturbed; (D) 5%, 10%, and 25% or more genes are perturbed; (E) 3 genes or 10% of genes are perturbed. Note that we use the third criterion to propose the set of genes to implement the knockouts experimentally.

**Supplementary Figure S24:** Growth curves of 10 mutant strains growth in M9 supplemented with different chemicals (SD calculated from 3 replicates).
