# Supplementary material for: An integrative, multi-scale, genome-wide model reveals the phenotypic landscape of Escherichia coli
Source: Mol Syst Biol. 2014 Jul 1;10(7):735. doi: 10.15252/msb.20145108 (PMC4299492; doi:10.15252/msb.20145108)
Supplement: Supplementary file 2 — Supplementary Table S1 [file msb0010-0735-sd2.pdf]

Supplementary Table S1 *E. coli* mutant strains

**Strain**	**Genotype**	**Source**	**M9 supplement**
MG1655	Wild type	Lab collection	As required
JW0195-1	F-, *Δ(araD-araB)567*, ***ΔmetN724****::kan*, *ΔlacZ4787*(::rrnB-3), *λ*-, *rph-1*, *Δ(rhaD-rhaB)568*, *hsdR514*	*E. coli* Genetic Stock Center	*L*-methionine
JW3911-1	F-, *Δ(araD-araB)567*, *ΔlacZ4787*(::rrnB-3), *λ*-, *rph-1*, *Δ(rhaD-rhaB)568*, ***ΔmetL727****::kan*, *hsdR514*	*E. coli* Genetic Stock Center	*L*-methionine
JW1733-1	F-, *Δ(araD-araB)567*, *ΔlacZ4787*(::rrnB-3), *λ*-, ***ΔastE742****::kan*, *rph-1*, *Δ(rhaD-rhaB)568*, *hsdR514*	*E. coli* Genetic Stock Center	*L*-arginine
JW2732-1	F-, *Δ(araD-araB)567*, *ΔlacZ4787*(::rrnB-3), *λ*-, ***ΔcysH764****::kan*, *rph-1*, *Δ(rhaD-rhaB)568*, *hsdR514*	*E. coli* Genetic Stock Center	*L*-cysteine
JW3331-2	F-, *Δ(araD-araB)567*, *ΔlacZ4787*(::rrnB-3), *λ*-, ***ΔcysG776****::kan*, *rph-1*, *Δ(rhaD-rhaB)568*, *hsdR514*	*E. coli* Genetic Stock Center	*L*-cysteine
JW3878-2	F-, *Δ(araD-araB)567*, *ΔlacZ4787*(::rrnB-3), *λ*-, *rph-1*, *Δ(rhaD-rhaB)568*, *ΔrhaT767::kan*, *hsdR514*	*E. coli* Genetic Stock Center	*L*-rhamnose monohydrate
JW3731-1	F-, *Δ(araD-araB)567*, *ΔlacZ4787*(::rrnB-3), *λ*-, *rph-1*, ***ΔrbsK781****::kan*, *Δ(rhaD-rhaB)568*, *hsdR514*	*E. coli* Genetic Stock Center	*D*-ribose
JW0740-3	F-, *Δ(araD-araB)567*, *ΔlacZ4787*(::rrnB-3), ***ΔgalK729****::kan*, *λ*-, *rph-1*, *Δ(rhaD-rhaB)568*, *hsdR514*	*E. coli* Genetic Stock Center	*D*-galactose
JW5628-1	F-, *Δ(araD-araB)567*, *ΔlacZ4787*(::rrnB-3), *λ*-, *rph-1*, ***ΔdgoA729****::kan*, *Δ(rhaD-rhaB)568*, *hsdR514*	*E. coli* Genetic Stock Center	*D*-galactose
JW2388-1	F-, *Δ(araD-araB)567*, *ΔlacZ4787*(::rrnB-3), *λ*-, ***ΔmntH729****::kan*, *rph-1*, *Δ(rhaD-rhaB)568*, *hsdR514*	*E. coli* Genetic Stock Center	Ferrous sulphate heptahydrate
