# Supplementary material for: An integrative, multi-scale, genome-wide model reveals the phenotypic landscape of Escherichia coli
Source: Mol Syst Biol. 2014 Jul 1;10(7):735. doi: 10.15252/msb.20145108 (PMC4299492; doi:10.15252/msb.20145108)
Supplement: Supplementary file 3 — Supplementary Methods [file msb0010-0735-sd3.pdf]

An integrative, multi-scale genome-wide model reveals the phenotypic landscape of *Escherichia coli*

Javier Carrera,[1,4] Raissa Estrela,[2] Jing Luo,[1] Navneet Rai,[1] Athanasios Tsoukalas,[1,3] Ilias Tagkopoulos,[1,3*]

[1]UC Davis Genome Center, University of California-Davis, Davis, CA, USA.
[2]Department of Molecular and Cell Biology, University of California, Berkeley, CA 94720, USA.
[3]Department of Computer Science, University of California-Davis, Davis, CA, USA.
[4]Present address: Department of Bioengineering, Stanford University, 318 Campus Drive, Stanford, California 94305, USA.
*Corresponding author.

Supplementary Methods

Contents

1. Construction of the *E. coli* gene expression compendium from high-throughput data sources 3
1.1 Microarray data sources 3
1.1.1 Affymetrix *E. coli* Antisense Genome Arrays. 3
1.1.2 Affymetrix *E. coli* Genome 2 Arrays. 3
1.2 Platform integration: ***E***. ***co****li* **M**icroarray **A**ffymetrix **C**ompendium (*Eco*MAC) 3
1.3 Gene expression variability analysis within *Eco*MAC 4
1.4 *Eco*Phe: A phenomics data compendium 5
2. Transcriptional interaction dataset for *E. coli* 6
2.1 Experimentally verified interactions. 6
2.2 Computationally inferred interactions 6
2.3 Correlation of gene expression between TFs and their target genes. 7
3. Construction of a signal transduction compendium for *E. coli* 8
3.1 The *Eco*ST database: Signal transduction mechanisms and sources 8
3.2 Signal transduction model 8
4. Integration of signal transduction and transcriptional models 10
4.1 Transcriptional model 10
4.2 Genetic and environmental perturbations. 11
4.3 Selection of nominal parameters that control gene expression of *E. coli*. 11
4.4 Prediction of gene expression under perturbations: Expression Balance Analysis (EBA) 12
4.4.1 Methodology description 12

4.4.2 Performance of genetic and environmental predictions. 13
5. Metabolic model 15
5.1 Flux analysis 15
5.2 Calculation of metabolic benefit under environmental perturbations 15
5.3 Calculation of metabolic benefit under genetic perturbations 16
6. Layer integration under a unifying model 18
6.2 Model Validation 18
7. Model optimization through targeted experimentation 20
7.1 Analysis of affected GO terms in *Eco*MAC 20
7.2 Model optimization through targeted experimentation 20
7.3 Experimental measurements 21
7.4 Comparison between experimental and predicted cell growth values 23

1. Construction of the *E. coli* gene expression compendium from high-throughput data sources

1.1 Microarray data sources

Microarrays rapidly became the preferred tool for high-throughput gene expression studies and although RNA-Seq will ultimately render the microarray technology obsolete, it is still used in some large-scale systems biology studies (Carrera et al, 2009; Carrera et al, 2012) and has the highest representation in gene expression data repositories (Faith et al, 2008; Yilmaz et al, 2011). Similar to previous work for *Escherichia coli*, *Shewanella oneidensis* and *Saccharomyces cerevisiae* (Faith et al, 2008) and extensions through the DREAM challenge for *Staphylococcus aureus* (Marbach et al, 2012), we have collected single-channel data from individual investigators, GEO, ArrayExpress and ASAP to create the largest gene expression compendium for *E. coli*. Importantly, we curated all experimental metadata included in our compendium from the respective publications, thus converting each chemical, strain, media, genetic modifications, and growth attributes into a structured and computable set of experimental features with consistent naming conventions and units.

1.1.1 Affymetrix *E. coli* Antisense Genome Arrays.

A total of 1,196 chips were downloaded from GEO (Platform ID: GPL 199). Microarray normalization was done using Robust Multi-chip Averaging (RMA) through the software RMAExpress. All raw data Affymetrix files (CEL files) were uploaded into RMAExpress and normalization was done as one batch. All arrays were background adjusted, quantile normalized, and probesets were summarized using median polish. Normalized data was exported as log-transformed expression values. Mapping of Affymetrix probeset ids to gene ids was done using the library files made available from Affymetrix. *Completion of these steps resulted in a total of 4,345 genes over the 1,196 microarrays*.

1.1.2 Affymetrix *E. coli* Genome 2 Arrays.

In total, 747 Affymetrix *E. coli* Genome 2 chips with available raw data were downloaded from GEO (Platform ID: GPL 3154) and compiled. Another 255 arrays that measured gene expression of rewired TRNs (Baumstark et al., 2013 – in submission) were added to the compendium. RMAExpress was also used here for RMA normalization. All arrays were background adjusted, quantile normalized, and probesets were summarized using median polish. Normalized data was exported as log-transformed expression values. Mapping of Affymetrix probeset ids to gene ids was done using R and the Bioconductor software packages to annotate *E. coli* genes with unique Entrez IDs. Control probesets and probesets that did not map unambiguously to one gene were removed. When multiple probesets were mapped to a single gene, expression values were averaged within each chip. *Completion of these steps resulted in a total of 4,291 genes over the 1,002 microarrays*.

1.2 Platform integration: ***E**. **co**li* **M**icroarray **A**ffymetrix **C**ompendium (*Eco*MAC).

Platform Integration: We integrated the results from (1.1.1) and (1.1.2) into a common database compendium, that we named *Eco*MAC. To do so, we only selected genes found in both platforms and we uniformly normalized the data by using microarray quantile normalization in Matlab R2012a as shown in **Suppl. Fig. 1A**. Completion of microarray normalization and gene filters resulted in *a compendium that consists of the expression of 4,189 genes over 2,198 conditions* collected from 127 scientific articles published from 114 different laboratories (see **Suppl. File 1**).

Transcription Factor and Enzyme annotation: We used two sources to define the list of potential transcription factors (TFs): (i) RegulonDB v8.1 (Salgado et al., 2013); and (ii), the DREAM5 dataset (Greenfield et al., 2010), where known and putative TFs were identified based on their Gene Ontology (GO) annotation. *A total of 328 genes were designated as potential TFs*. Next, we used a recent study focused on the *E. coli* metabolism (Orth et al., 2011) to identify all genes catalyzing metabolic reactions. *We identified 1,357 enzymes in our gene list*, four of which were also categorized as TFs (*putA*, *alaS*, *pepA* and *nadR*).

The microarray compendium and gene list are supplied in the **Suppl. File 1**. In addition to the gene expression data, **Suppl. File 2** contains the following attributes: (a) database accession number or source ID, (b) author and date information, (c) strain information, (d) medium and treatment information, (e) temporal information for the experiment (time series, phase of growth), (f) experiment type (knockout, overexpression, rewiring, reshuffling). Raw CEL files can be downloaded upon request for further processing.

Strain and condition distributions: The *Eco*MAC compendium includes data from 31 strains and 15 mediums (**Suppl. Fig. 2** and **Suppl. Fig. 3**, respectively). From the 2,198 arrays, 718 arrays correspond to genetic perturbation experiments (knockout, overexpression, rewiring, reshuffling) and 332 arrays correspond to environmental perturbations (variation in carbon, nitrogen, phosphate, or metal sources, aerobic and anaerobic conditions, or supplemented media), while the rest of the 1148 arrays come from a high diversity source of experiments to study biofilm formation or microbial adaptive evolution. From the 31 strains that are present in the *Eco*MAC compendium, the MG1655, BW25113 and EMG2 strains are the most prevalent with more than 76% of arrays present (**Suppl. Fig. 2**). Similarly, from the 15 medium types, LB and M9 were over-represented, with more than 85% of arrays (**Suppl. Fig. 3**). We have identified 90 arrays that contain the gene expression of MG1655 *E. coli* cells at the exponential and stationary phase that were grown in aerobic conditions, M9 salt, LB media, and 2g/L glucose as the sole carbon source. In this study, we have defined these conditions to be the wild type (WT) or "ground" conditions, while experimental settings that deviate from this configuration are classified as environmental perturbations (332 arrays). We found 518 arrays where the genetic perturbations were imposed on TFs or enzymes (437 and 81 arrays, respectively, **Suppl. Fig. 1B**).

Next, we quantified the number of genes perturbed in at least one array characterized by having genetic or environmental perturbations in *Eco*MAC. Interestingly, we found 141 different genes altered by accounting gene knockouts, gene over-expression, TF rewirings and TFs directly affected by different environmental perturbations. **Fig. 3A** depicts all TF perturbations presented in *Eco*MAC. The cluster coefficient of the TRN is 0.122, with 21 connected components, diameter equal to 8 links, a characteristic path length of 2.871 links, and an average number of 4.494 TFs regulating all genes.

1.3 Gene expression variability analysis within *Eco*MAC

We analyzed the gene expression diversity across the different conditions contained in the *Eco*MAC compendium. For this purpose, we used two similarity metrics that describe the distance between each array and the gene expression corresponding to the WT conditions. First we calculated the relative error between the target (y^c) and WT (y^{WT}) gene expression profile for condition c, as follows:

$$e_c = \frac{1}{N_g} \sum_{N_g} \left| \frac{y_g^c - y_g^{WT}}{y_g^{WT}} \right| \qquad (1),$$

where N_g is the number of genes in the *Eco*MAC and y_g^{WT} is the gene expression of a WT array. Notice that we defined a pseudo-array as the gene expression average of all WT arrays. y_g^{WT} was selected by minimizing the distance with respect to the pseudo-array. Similarly, we used the Pearson correlation coefficient (*PCC*) given by $P_c = \rho(y_g^c, y_g^{WT})$ as a second measure of diversity. High values of e_c and low values of *PCC* imply high deviation in gene expression with respect to the wild-type pattern, and vice versa for low e_c and high *PCC* values (**Suppl. Fig. 4**).

1.4 *Eco*Phe: A phenomics data compendium

We identified 616 arrays in *Eco*MAC, where the bacterial growth rate is reported in the corresponding scientific articles, with growth rate values ranging from 0.09 to 2.14 h^{-1} (**Suppl. Fig. 1**). More than 84% of those arrays were implemented in MG1655 strain, and 27% and 60% were measured on M9 and LB media respectively. Additionally, 28% of the arrays were measured in different times of experiments related to time series, 14% were related to studies in evolution, and 54% of the arrays were measured in the exponential or mid-exponential phase of growth.

2. Transcriptional interaction dataset for *E. coli*

2.1 Experimentally verified interactions.

We used all interactions reported in RegulonDB v8.1 that were experimentally validated to support the existence of regulatory interactions. After removing those interactions that included genes that are not present in *Eco*MAC, we compiled a list of 3,704 regulatory interactions, 115 of which were auto-regulatory interactions (3.1%) (**Suppl. File 3**). Positive interactions are slightly more represented than negative interactions (1,807 vs. 1,664), with 233 interactions being dual in nature. Then we created 3 sets of data based on the confidence level of the included interactions: 1. A first set with 566 "confirmed" evidence interactions (at least two independent types of experimental validation). 2. A second set that includes all 566 confirmed, and another 2,517 "strong" evidence interactions where a low throughput high confidence method was used (e.g., foot-printing, site mutation, protein binding) for a total of 3,083 interactions. 3. A third set that includes all 3,704 interactions, with 711 of them based only on "weak" evidence (RNA-Seq, ChIP-Seq).

2.2 Computationally inferred interactions

We used the results from Marbach et al. (Marbach et al, 2012) that provide 1,468 interactions identified with high precision (TP/TP+FP >0.5) by 35 network inference algorithms that were applied to 805 arrays (that constitutes 37% of arrays in *Eco*MAC) over 487 different experimental conditions.

In addition, we built a consensus network from *Eco*MAC by using the three highest ranked inference algorithms that were a part of the DREAM5 challenge (Marbach et al, 2012), namely the Inferelator (Greenfield et al., 2010), GENIE3 (Huynh-Thu et al., 2010), and TIGRESS (Haury et al., 2012) methods. Inclusion of the CLR (Faith et al., 2007) and ANOVA methods (Küffner et al., 2012) decreased the predictive ability of the meta-classifier, possibly due to the lack of reference arrays for ANOVA and the fact that Inferelator already incorporates an extended version of CLR as a pre-processing step. Ranking of the putative interactions in the meta-classifier was performed equal to Marbach et al. (Marbach et al, 2012), where the rank r of interaction i over N inference methods are given by $Rank_i = \frac{1}{N}\sum_{j=1}^{N} r_j(i)$.

Two gold standards were used from RegulonDB v8.1, one that includes only 566 confirmed interactions (i.e. interactions that have at least two strong evidence types) and another that includes 3,083 strong interactions. For reference, we used the dataset used in the DREAM5 evaluation as the third gold standard (2,066 connections with mixed strong and weak evidence based on RegulonDB v6.8). We compared the results of the community network generated by the 3 or 35 methods over these three testing datasets. All the datasets and a ranked list of the new top connections (not included in the golden standards) in the top ranked 365 inferred connections that correspond to 0.5 precision, can be found in **Suppl. File 4.** The comparison results are presented in **Suppl. Fig. 5.**

It is evident in **Suppl. Fig. 5E-F** that the inference using the *Eco*MAC dataset gives better results than using the DREAM5 arrays, both with the consensus/ensemble techniques and the 3 individual methods. Figures 5A-D show the performance of the inference against two golden standards, one containing the confirmed RegulonDB v8.1 connections (A-B) and the other containing the strong connections (C-D). The performance in ROC is better than the DREAM5 equivalents.

The parameters used for each algorithm follow in summary: (a) *GENIE3*, Tree method: Random Forests; number of randomly selected variables at each node of a tree: square root of the number of transcription factors, sqrt(328) = 18; number of trees grown in an ensemble: 1000; (b) *TIGRESS*, number of bootstraps: 100; randomization parameter alpha for stability selection in [0,1]: 0.2; number of LARS steps at each iteration of stability selection: 5; scoring method: "area"; (c) *Inferelator*, maximum number of regulators for each target gene: 30; number of bootstraps performed: 1; time scale in which regulatory interactions take place (τ): 10. The top 100,000 predictions were used for evaluation from each method. The GO term and enrichment analysis was performed in Cytoscape, with the Bingo package and in DAVID v6.7. The enriched categories and the corresponding genes are in **Suppl. File 4**. From the top 500 computationally inferred interactions (that corresponds to Precision 0.45, 0.13 recall-TPR and 0.005 FPR on the meta-classifier; **Suppl. Fig. 3C**), the most enriched biological processes are response to stimulus (169 interactions), response to external stimulus (89 interactions), locomotion - locomotory behavior - taxis (81 interactions), and cell motility - localization of cell - ciliar or flagellar motility (33 interactions) (**Fig. 3D**).

2.3 Correlation of gene expression between TFs and their target genes.

We used the *PCC* as a measure of correlation between the expression profiles of the TFs and their targets (experimental and inferred interactions), and compared the results to a null model of random TF-gene pairs. We performed an analysis for the experimentally-validated interaction set (3,704 interactions, **Suppl. Fig. 6A** and **6C**), and for the experimentally-validated plus computationally-inferred interactions (5,172 interactions, **Suppl. Fig 6B** and **6D**). In both cases, candidate TF-gene pairs exhibit levels of similarity in expression significantly higher than random TF-gene pairs (Kolmogorov-Smirnov test $p < 10^{-10}$ and Mann-Whitney test $p < 10^{-10}$). We also computed the slope for both lognormal *PCC* distributions (with $p < 10^{-10}$), with a higher slope found in the random case.

3. Construction of a signal transduction compendium for *E. coli*

3.1 The *Eco*ST database: Signal transduction mechanisms and sources

The inclusion of a signal transduction model is essential to capture the information flow both between the environment and cellular mechanisms, as well as among cellular components. In this work, we focused on the signal transduction as it pertains to gene regulation of TF genes. To that end, we curated various databases (EcoCyc (Keseler et al, 2012) and RegulonDB (Salgado et al, 2013)) and more than 150 publications to identify 151 instances of signal transduction systems (STSs) where the expression level of one or more TFs is regulated by the presence of effector molecules (**Suppl. Fig. 7A**). Interestingly, 71 of these TF-effector interactions fall under one of the following four types of auto-regulation: (a) Type I (28 instances): the TF represses its own expression in the absence of an inducer, while de-repression occurs at its presence (e.g., *lldR* and *L*-lactate; **Suppl. Fig. 7A and Suppl. Fig. 8A**), (b) Type II (11 instances): the TF represses its own expression in the presence of the effector (e.g., *fur* and iron; **Suppl. Fig. 7B** and **Suppl. Fig. 8A**), (c) Type III (4 instances): two component systems where a histidine kinase sensor is auto-phosphorylated in the presence of an effector and transfers the phosphate to the actual TF that can in turn positively (3 instances) or negatively (1 instance) regulate its own expression (e.g., *dpiA* and citrates **Suppl. Fig. 7C**) and (d) Type IV (28 instances) where the effects in the gene expression of the TF are empirically observed in presence of the effector but the corresponding mechanism is not clear (e.g., *fhlA* and formate; **Suppl. Fig. 7E**). The remaining 80 signal-mediated regulatory interactions were described in literature, but they did not show a significant change in gene expression levels in presence of the effectors. **Suppl. File 5** contains information on all the signal transduction systems that we considered.

3.2 Signal transduction model

To model the effect of STSs on gene expression, we considered both the case where the effector concentration impacts the abundance of the corresponding TF, and the case where the effector binds to the TF and alters its structural conformation and functionality. In the first case, we defined a linear constraint to describe the TF expression, y_{TF}, as a function of changes in environmental signals, Δn_E:

$$y_{TF} = y_{TF}^{\mathrm{wt}} + \Omega\left(C_{TF}^{\max} - C_{TF}^{\min}\right)\chi_{TF}^{E}\frac{\Delta n_E}{\Delta n_E^{\max}} \qquad (2),$$

where y_{TF}^{wt}, C_{TF}^{min}, and C_{TF}^{max} are the wild-type, minimum, and maximum expression values of the TF gene obtained from *Eco*MAC. Δn_E^{max} (effector strength) is an empirical parameter that characterizes the levels of environmental signals (mmol/gDW) where the TF expression reaches its minimum or maximum level. Δn_E is the change in concentration of the environmental signal, $(n_{ES} - n_{ES}^{wt})$, from the reference levels (WT environment). Similarly, χ_{TF}^{E} is a parameter that represents whether the TF expression increases ($\chi_{TF}^{E} = 1$) or decreases ($\chi_{TF}^{E} = -1$) when the effector is present. The global parameter Ω was used to fine-tune the STSs, and more information how it is trained can be found in Section 4.1.

In the second case, we modeled the change in the TF activity after it is bound by an effector *g*, by introducing a binary variable τ_{TF}^{g} that had the value one when the TF was still functional after the binding event ($\tau_{TF}^{g} = 1$) and value zero otherwise ($\tau_{TF}^{g} = 0$). The values of this binary variable were determined through literature search and curation of the RegulonDB database.

As an example consider *lldR*, a well-known TF that is activated by environmental signal *L*-lactate. As with all STSs that we consider here, we assume that (*i*) the minimum value of the TF expression (in this case, $y_{lldR}^{min} = y_{lldR}^{wt}$) is reached when the concentration of the environmental signal, *L*-lactate, is minimal ($n_{L-lactate} = n_{L-lactate}^{min} = 0$ M); (*ii*) the maximum level of the TF expression (y_{lldR}^{max}) is related to the highest concentration of *L*-lactate ($n_{L-lactate}^{max} = 10$ mM), given a saturated TF concentration. Hence, we could estimate $\Delta n_{L-lactate}^{max} = n_{L-lactate}^{max} - n_{L-lactate}^{min} = 10$ mM and, $\chi_{lldR}^{L-lactate} = 1$. **Suppl. File 5** contains values for Δn_{E}^{max}, χ_{TF}^{E}, and τ_{TF}^{g} parameters for each TF-effector combination.

From the 151 STSs, 53 of them were described by having a transcriptional auto-activation ($\chi_{TF}^{E} = 1$), 16 showed an auto-repression ($\chi_{TF}^{E} = -1$), and 32 interactions did not show variation in the TF expression ($\chi_{TF}^{E} = 0$). The remaining 50 interactions have not been conclusively investigated. Furthermore, 93 of the 151 interactions between TFs and effectors were reported to suppress ($\tau_{TF}^{g} = 0$; 36 instances) or induce ($\tau_{TF}^{g} = 1$; 57 instances) their regulatory activity. **Fig. 3B** depicts the signal transduction associations with the different transcription factors (416 transcriptional interactions between 183 interconnected TFs and 19 non-TF genes), and **Supp. Fig. 9** summarizes the distribution of the signal transduction systems as a function of the effector strength (Δn_{E}^{max}).

STS association to environmental perturbations: To investigate the coverage of various environmental stimuli by the STSs, we calculated their occurrence frequency in the following 7 environmental sources: 7 distinct acids (related to 7 STSs), 26 carbon sources (35 STSs), 11 nitrogen sources (13 STSs), 19 metal sources (34 STSs), oxygen (5 STSs), 7 phosphorous sources (12 STSs), and 37 supplements such as amino acids or their precursors (45 STSs).

4. Integration of signal transduction and transcriptional models

4.1 Transcriptional model

The mRNA dynamics of all genes in the compendium (vector $\bar{y}_g$) as a function of the TF concentration (vector $\bar{y}_{TF}$) is given by equation (3):

$$\begin{aligned} &\frac{d}{dt}\bar{y}_g = \bar{a} + \bar{\bar{b}}\,\bar{y}_{TF} - \bar{\bar{\xi}}\,\bar{y} \\ &\phi^{-1}\,\bar{y}_g^{\min} \le \bar{y}_g \le \phi\,\bar{y}_g^{\max} \end{aligned} \qquad (3),$$

where $\bar{a}$ is a vector of the basal transcription coefficients, $\bar{\bar{b}}$ is a matrix with elements b_{ij} that represent the effect of the jth TF to the ith gene, and $\bar{\bar{\xi}}$ contains the degradation and dilution rate constants for each gene. The maximum ($\bar{y}_g^{max}$) and minimum ($\bar{y}_g^{min}$) values of gene expression for each gene were obtained from *Eco*MAC. The parameter ϕ, which is only positive, alters the bounds for the expression for each individual gene. It allows for the removal of outliers (when $0 < \phi < 1$) and also for imposing smaller/greater values than the ones in the compendium (when $\phi > 1$), i.e., several arrays will not be taken into account for some specific genes. Here, we set $\phi = 0.9$ in which case the domain for each gene includes the values for more than 95% of the arrays in *Eco*MAC (**Suppl. Fig. 10**). In steady-state equation (3) becomes:

$$\begin{aligned} &\bar{y}_g = \bar{\alpha}' + \bar{\bar{\beta}}\,\bar{y}_{TF} \\ &\bar{C}_g^{\min} \le \bar{y}_g \le \bar{C}_g^{\max} \end{aligned} \qquad (4),$$

where $C^{min} = \phi^{-1}y^{min}$ and $C^{max} = \phi y^{max}$ denote the min and max expression capacities. We trained our model first with the 3,704 transcriptional interactions verified experimentally (i.e., the cases where $\beta_{TF}^{g} \neq 0$). For that, we performed linear regression analysis to train equation (3) by using the gene expression profiles obtained from *Eco*MAC. Additionally, we constrained the linear regression problem by imposing positive ($\beta_{TF}^{g} > 0$) or negative ($\beta_{TF}^{g} < 0$) regulatory coefficients to be consistent with the transcriptional activations or repressions that are confirmed in RegulonDB (566 regulations with confirmed interactions with 2 or more types of experimental validation; see Section 3.1). After training the model parameters, we compared the regulatory effect (activation, repression) inferred by our model for the interactions categorized in RegulonDB with strong but not confirmed evidence (2,517 candidates; see Section 3.1 for definitions) with a *PCC* more than 0.5 (367 interactions) and 0.7 (154 interactions) (**Fig. 3C**). To assess the predictive ability of our model, we calculated the precision-recall curves for both activation and repression. The area under the curve A was found to be significantly higher from the random model for those interactions with *PCC* > 0.50 (A_{act} = 0.650 and A_{repr} = 0.668; **Suppl. Fig. 11A**) and the predictive power of the model increases significantly for interactions with *PCC* > 0.70 (A_{act} = 0.743 and A_{repr} = 0.905; **Suppl. Fig. 11A**). Based on our analysis, activatory interactions are more difficult to predict than their inhibitory counterparts (**Suppl. Fig. 11A**), and the predictive power of the model increases proportionally with the correlation between the TF and target profiles, both for activations and repressions (**Suppl. Fig. 11B**).

Next, the set of equations in (3) was split into two subsets, based on whether the candidate gene is a TF (328 cases) or a non-TF (3861 cases):

$$\bar{y}_{TF} = \bar{\alpha} + \bar{\bar{\beta}}\, \bar{y}_{TF}$$
$$\bar{C}_{TF}^{\min} \le \bar{y}_{TF} \le \bar{C}_{TF}^{\max} \quad (5),$$

$$\bar{y}_{g} = \bar{\alpha} + \bar{\bar{\beta}}\, \bar{y}_{TF}$$
$$\bar{C}_{g}^{\min} \le \bar{y}_{g} \le \bar{C}_{g}^{\max} \quad (6).$$

To calibrate TF expression, the newly redefined constitutive transcription rate in (5) included a perturbative term (e) that fits only the TF expression profile for the defined optimal (WT) condition (y^{WT}): $\bar{\alpha} = \bar{\alpha}' + \bar{e}$ where $\bar{e} = \left(1 - \bar{\bar{\beta}}\right)\bar{y}_{TF}^{WT} - \bar{\alpha}$. Hence, the error for the TF expression prediction in the WT condition will be zero.

We also compared the distributions of regulatory (β) and basal (α) transcription coefficients between the two transcriptional models constructed (see equations (5) and (6)) by using only (*i*) experimental interactions, and (*ii*) both experimental and inferred regulations. For our purpose, we used Mann-Whitney tests ($P < 10^{-39}$) to evaluate differences in the location, and Kolmogorov-Smirnov tests ($P < 10^{-34}$) to assess the difference in shape of the inferred parameter distributions between the two transcriptional models mentioned previously. Highly significant differences were found between the regulatory (**Suppl. Fig. 12A**) and basal (**Suppl. Fig. 12B**) coefficient distributions of models including purely experimental, or both experimental and inferred interactions. Moreover, we sought activators and repressors inferred by our two models. For that, we defined a minimal regulatory capacity (β_{th}) above which we categorized the interactions of TFs and their targets as activatory ($\beta > \beta_{th}$) or inhibitory ($\beta < \beta_{th}$). **Suppl. Figs. 12C** and **12D** show the number of activations and repressions inferred in our models, respectively, as well as the number of regulations experimentally verified. Interestingly, our results, when $\beta_{th} < 0.2$, support the "Demand theory" (Savageau, 1998), which proposed a global ratio between repressions and activations of 0.5 (**Suppl. Fig. 12E**).

4.2 Genetic and environmental perturbations.

To predict gene expression under genetic (over-expression, gene knock-out, gene reshuffling, TF rewiring) and environmental (uptake of metabolic/chemical compounds) changes, we explicitly defined transcriptional and environmental constraints. Genetic perturbations of a set of genes, $g \in G$ are modeled by modifying its gene expression y_g as follows:

Over-expression gene: $y_g = C_g^{max}$ (7),

Gene knockout: $y_g = C_g^{min}$ (8),

Gene rewiring: $y_g = \alpha + \alpha^P + \sum \beta\, y_{TF} + \sum \beta^P y_{TF}^P$ (9),

Gene reshuffling: $y_g = \alpha^P + \sum \beta^P y_{TF}^P$ (10)

where index P denotes the promoter that drives gene g in the rewired/reshuffled circuit.

Environmental perturbations in terms of changes in the intra-/extra-cellular metabolic fluxes (v) or in the concentrations of chemical compounds (IPTG, arabinose, etc.) are included as constraints in equation (2) (see Section 2.2).

4.3 Selection of nominal parameters that control gene expression of *E. coli*.

Model parameters can be categorized as local or global. Local parameters, such as the basal or regulatory coefficients for transcription, are optimized based on the procedures described in Section 4.1. We also have two global parameters: a parameter defining the boundary conditions

of gene expression (ϕ) and a parameter that provides a fine-tuning of all signal transduction systems (Ω). As discussed in Section 4.1, we chose $\phi = 0.9$ so that the minimum and maximum values of the gene expression capacities are reduced by 5% which translates that 4.9% of the arrays were not considered to define gene expression variability (**Suppl. Fig. 10**). Hence, specific conditions of the compendium related with global cellular stresses were not considered, because they exhibited high variations in their expression profiles. Since our model is able to predict only small/local perturbations and not global perturbations (heat shocks, pH variations), we imposed boundary conditions lower than the values observed in the whole compendium. Similarly a value of 1 was chosen for Ω. From sensitivity analysis we performed, the predictive power of our model is largely insensitive – i.e., 2.30% ($p > 0.048$) of mean variation in the number of arrays well predicted –, up to high perturbations of these parameters ($\phi \in (0.9, 1.5)$ and $\Omega \in (0.5, 1.5)$) (**Suppl. Fig. 13**).

4.4 Prediction of gene expression under perturbations: Expression Balance Analysis (EBA)

4.4.1 Methodology description.

We developed a novel method called "Expression Balance Analysis" (EBA) to predict the global gene expression of *E. coli* under genetic modifications and environmental changes. We have formulated an optimization problem to find the gene expression profile (y_g) that accomplishes four sets of constraints (phenomenological, capacity, environmental, and genetic). Following an approach of minimizing the change to the WT state following a perturbation that is widely used in genome-scale modeling (Segre et al., 2002), we used a fitness function, *E*, that minimizes the gene expression errors of the 328 TFs (ε_{TF}):

Minimize: $$\varepsilon_{TF} = \bar{y}_{TF} - \bar{\alpha} + \bar{\bar{\beta}}\, \bar{y}_{TF} \qquad (11),$$

subject to constraints imposed by the environment and the genomic modifications. In a matrix form, (11) is formulated as $\bar{\bar{\Sigma}} \begin{bmatrix} \bar{y}_{TF} \\ \bar{\varepsilon}_{TF} \end{bmatrix} = \bar{\alpha}$, where $\Sigma = \begin{bmatrix} \mathrm{Id} - \bar{\bar{\beta}} & \mathrm{Id} \end{bmatrix}$. Then we constructed a quadratic programming problem where the variables are a vector of 656 components containing the TF expression profiles and their corresponding model errors:

Minimize: $$E = \frac{1}{2}\begin{bmatrix} \bar{y}_{TF} & \bar{\varepsilon}_{TF} \end{bmatrix} \bar{\bar{H}} \begin{bmatrix} \bar{y}_{TF} \\ \bar{\varepsilon}_{TF} \end{bmatrix} + \bar{f} \begin{bmatrix} \bar{y}_{TF} \\ \bar{\varepsilon}_{TF} \end{bmatrix} \qquad (12),$$

subject to: $\bar{\bar{\Sigma}} \begin{bmatrix} \bar{y}_{TF} \\ \bar{\varepsilon}_{TF} \end{bmatrix} = \bar{\alpha}$ (13), phenomenological constraints;

$$\bar{y}_{TF} \geq \bar{C}_{TF}^{\min} \\ \bar{y}_{TF} \leq \bar{C}_{TF}^{\max}$$ (14), capacity constraints;

$$\bar{y}_{TF} = F_G\left(\bar{C}^{\min}, \bar{C}^{\max}, \bar{\alpha}, \bar{\beta}, \bar{y}_{TF}\right)$$ (15), genetic constraints;

$$\bar{y}_{TF} = F_E^{(1)}\left(\bar{C}^{\min},\ \bar{C}^{\max},\ \bar{y}_{TF}^{wt},\ \chi_{TF}^{E},\ \Delta n_E^{\max},\ \Delta n_E\right)$$
$$\bar{y}_g = F_E^{(2)}\left(\bar{\alpha},\ \bar{\beta},\ \bar{y}_{TF}\cdot\bar{\tau}_{TF}\right)$$
(16), environmental constraints.

The hessian matrix is $H = \begin{bmatrix} \bar{\bar{0}} & \bar{\bar{0}} \\ \bar{\bar{0}} & \bar{\bar{I}} \end{bmatrix}$, and $\bar{f} = \bar{0}$. The F_G function represents all rules described in the equations (7-10). The function $F_E^{(1)}$ represented in the equation 2 is used to impose the environmental restrictions in TF expression. The function $F_E^{(2)}$ was represented in the equation 5 by replacing y_{TF} by $y_{TF} \cdot \tau_{TF}$ to simulate the environmental constraints also described in Section 2 for non-TF genes. Then we use quadratic programming to solve EBA, and consequently, we obtain the expression profile of all TFs (y_{TF}). After that, we use the equation 6 to compute the entire gene expression profile (i.e., non TFs).

4.4.2 Performance of genetic and environmental predictions.

To assess the predictive power of our transcriptional model, we used a testing set of arrays from *Eco*MAC with genetic modifications (gene knockouts, over-expressions and rewired TRNs; 437 arrays total) and environmental perturbations (55 arrays; see **Suppl. Fig. 1B**). For each array, we computed the pearson correlation coefficient between the predicted ($\bar{y}$) and experimentally measured ($\bar{y}^{\exp}$) expression profiles as *PCC*= $P(\bar{y}, \bar{y}^{\exp})$). Hence, *PCC* quantifies the performance of our method to predict genetic/environmental perturbations of a given array. Note that $\bar{y}$ is predicted using EBA (Section 4.3). We compared the performance of our EBA model with respect to the following randomized models:

1) Random selection of expression profiles from *Eco*MAC ($\bar{y}^{Eco\mathrm{MAC}}$), with a performance given by $PCC_1 = \bar{P}_1(\bar{y}^{Eco\mathrm{MAC}}, \bar{y}^{\exp})$.
2) Wild-type expression profiles ($\bar{y}^{\mathrm{WT}}$) predicted by EBA without imposing any genetic/environmental constraints in equations (15-16), which results to a performance of $PCC_2 = \bar{P}_2(\bar{y}^{\mathrm{WT}}, \bar{y}^{\exp})$ for each profile. These profiles were generated by running EBA with different perturbation terms (i.e., different optimal conditions, y^{WT}, selected from *Eco*MAC; see section 1.2), thus affecting the basal expression terms in equation (5).
3) Expression profiles predicted by EBA by imposing random genetic and environmental constraints (equations 15-16) ($\bar{y}^{\mathrm{rand}}$), $PCC_3 = \bar{P}_3\left(\bar{y}^{\mathrm{rand}}, \bar{y}^{\exp}\right)$ represent the predictive power of the null-model.

Next, we used two criteria to assess the predictive power of each array included in the testing set. First, we evaluated how close is the predictive power of EBA with respect to the predictive power distribution of a null-model. For that, we computed the statistic *Z*-score:

$$Z_i = \frac{PCC - \langle PCC_i \rangle}{\sigma_{PCC_i}} \qquad (17),$$

where i is one of the three null-models previously described. Hence, we defined a ratio of *well-predicted arrays* across the testing set as those where $Z_i > 3$ ($p < 10^{-3}$). Hence, to assign an array predicted by EBA (where *PCC* is its predictive performance) as a well-predicted condition by the first null-model (PCC_1):

$$PCC > 3\ \sigma_{PCC_1} + \langle PCC_1 \rangle \qquad (18).$$

Analogously for the second and third null-model (i.e., EBA-guided random models):

$$PCC > 3\ \sigma_{PCC_2} + \langle PCC_2 \rangle \qquad (19),$$

$$PCC > 3\ \sigma_{PCC_3} + \langle PCC_3 \rangle \qquad (20).$$

We analyzed the predictive power by computing *PCC* across the entire gene expression profile (global scores) or also selecting only a specific set of genes (local scores) (**Suppl. Fig. 14A** and **14B**). In the second case, we considered local genes, defined as those located with a distance of two links/hops from the specific gene perturbed in the array. Note that the TFs altered in an environmental perturbation are the set of genes affected directly by some environmental effector described in Section 2.

To define an overall score to assess the EBA performance, we added a second criterion to characterize well-predicted arrays as those in which the *PCC* was higher than a threshold. Note that we used the average of all *PCCs* as threshold. **Fig. 4A** illustrates the overall scores as the ratio of well-predicted arrays that contained genetic and/or environmental perturbations.

We also used another scoring function to evaluate the consistency of predictions in EBA. To measure this, we computed the relative error between the predicted and experimental expression profiles (**Suppl. Fig. 14C** and **14D**). In addition, we tested EBA by training the transcriptional model with both experimental and inferred regulations. We evaluated EBA's capacity to predict different types of genetic perturbations such as gene knockouts, over-expression and rewired TRNs (**Suppl. Fig. 15**).

In addition, we studied the performance of EBA by training random sub-sets of transcriptional interactions or by excluding random sets of experimental and inferred interactions with different sizes (**Suppl. Fig. 16A** and **16B**). We also performed a 5-fold cross-validation to investigate the robustness of EBA by using different training subsets of *Eco*MAC (**Suppl. Fig. 16C** and **16D**).

5. Metabolic model

5.1 Flux analysis

Flux calculation: We used Flux Balance Analysis (FBA) (Orth et al, 2011) to predict the flux values so that the metabolic benefit (or biomass variation) is maximized. Mathematically, the problem is represented in the following way:

$$\text{Maximize} \quad B = c^T v \qquad (21).$$

$$\text{subject to:} \quad \begin{array}{l} S\,v = 0 \\ V_{\min} \leq v \leq V_{\max} \end{array}$$

The flux through all the reactions in the network was represented by the vector *v; c* is a vector of weights indicating how much each reaction contributes to the benefit. This model considers 324 exchange reactions and the internal reactions are catalyzed by a set of 1,357 enzymes.

Calculation of flux bounds: We used the *E. coli* metabolic model iJO1336 (Orth et al, 2011) to perform flux variability analysis (FVA) (Mahadevan et al, 2003) to calculate the bounds on the reaction fluxes. This method uses constraint-based modeling to minimize the flux space (V) for the identification of minimal reaction sets. The mathematical formulation follows as:

$$\text{Maximize:} \quad V_i \qquad (20),$$

$$\text{subject to:} \quad \begin{array}{l} S\,V = 0, \\ f^T\,V = B_{opt} \\ LB \leq V \leq UB \end{array}$$

where B_{opt} is the value of a supplied growth rate or metabolic benefit in optimal conditions, S is the stoichiometric matrix defined by 2,583 metabolic reactions and 1,805 metabolites, f is a vector containing the stoichiometric coefficients of growth-related reactions, and LB and UB are the metabolic flux bounds. This linear programming (LP) problem is computed for every metabolic flux in the model. We used fastFVA (Gudmundsson and Thiele, 2010), which is a Matlab implementation of FVA, to calculate V_{min} and V_{max} of all metabolic reactions. Note that we considered all flux solutions that achieved an optimal and suboptimal growth rate. Lower bounds on B were set to 50% optimal metabolic benefit (B_{opt} obtained in the WT condition) by using the FVA algorithm with the transcriptional constraints that were provided by the transcriptional model.

5.2 Calculation of metabolic benefit under environmental perturbations

In order to test the metabolic model under various environmental conditions, we simulated 100 random environments where cells grew in minimal media and a growth-affecting parameter in abundance or limitation (carbon sources, nitrogen, supplemental amino acids, or metals). **Suppl. File 6** contains a full description of the environmental sources used. In all cases, the environment contained O_2, CO_2, H_2O, SO_4, and PO_4, with fluxes set at 5 $gDW^{-1}\,h^{-1}$. **Suppl. Fig. 17** depicts the change in growth rate as a function of source availability. In all cases, the model provides a quantitative measure of the growth rate increase for the different environmental perturbations.

5.3 Calculation of metabolic benefit under genetic perturbations

We explored the change in the growth rates in the case of genetic perturbations (knockout, over-expression) for the following cases: (a) enzymes related to growth and (b) transcription factors that modulate expression of enzymes. For the later, we used both direct (i.e. enzyme is directly regulated by a TF) and indirect interactions between TFs and enzymes. For this analysis, we used the TRN based on experimental interactions.

We created a novel method called **Tra**nscription-based **M**etabolic Flux **E**nrichment (TRAME) to integrate metabolic and transcriptional regulatory networks modifying the V_{min} and $V_{\max}$ calculated from FVA for each metabolic flux. This approach changes the values of the flux bounds by multiplying V_{min} and $V_{\max}$ by the probability that the enzymes are expressed (*P*-function) as in the WT condition. This function integrates all enzymes catalyzing a specific metabolic reaction.

More specifically, we define the *P*-function for a given metabolic reaction catalyzed by the enzyme e, as the scaled ratio between the predicted ($\hat{y}_e$) and wild-type (y_e^{WT}) enzyme expression:

$$P_e = \left(\frac{\hat{y}_e}{y_e^{\mathrm{WT}}} \right)^n \qquad (22),$$

where $n = 2$ is a parameter that allows to factor in the variability observed on the wild-type arrays regarding the expression of that specific enzyme, y_e^{WT}. Hence, for those enzymes where the expression levels predicted are higher than the wild-type, the corresponding P_e function will be higher than zero, and consequently, the boundary conditions for the metabolic fluxes ($P_e\, V_{min}, P_e\, V_{max}$) will be higher. Note that for metabolic reactions in which several enzymes are governing, we will define a *P*-function generalized based on the logic activity of those enzymes. For instance, one specific metabolic reaction catalyzed by three enzymes A, B, and C that perform the following logic function: (A ***OR*** (B ***AND*** C)); the *P*-function is defined as:

$$P_{generalized} = \mathrm{average}\left(\frac{\hat{y}_A}{y_A^{\mathrm{WT}}}, \min\left(\frac{\hat{y}_B}{y_B^{\mathrm{WT}}}, \frac{\hat{y}_C}{y_C^{\mathrm{WT}}} \right) \right) \qquad (23).$$

Hence, OR *vs* AND logic gates are replaced by the average or minimum-functions respectively, and the assumption is that each enzyme acts independently and equally. This transformation provides new boundary conditions of all metabolic reactions for the equation 21, as follows:

$$P\, V_{\mathrm{min}} \le v \le P\, V_{\mathrm{max}} \qquad (24).$$

By using this representation, we can use these new upper and lower bounds in FBA to predict a growth rate after genetic perturbations.

We used the model to predict phenotypic variations of the benefit function under genetic perturbations of growth-related enzymes and TFs. We used the random environmental conditions as in Section 5.2. For all environments tested, all exchange fluxes were set to V_{min} = 0.1 $\mathrm{gDW}^{-1}\,\mathrm{h}^{-1}$ to increase the sensitivity of the model to small-scale effects throughout all reactions. As an approximation, for a metabolic reaction described by only one enzyme, A, we could simulate knockout and overexpressed enzymes by defining the *P*-function as follows:

1) $P_A = 0$ represents that A is knockout enzyme and consequently, will impose its metabolic flux equal to zero.
2) $P_A = 1$ the enzyme is expressed to conduct the reaction in wild-type manner and consequently, the flux bounds are unaffected by the P-function.
3) $P_A > 1$ to represent that A is over-expressed enzyme for that reaction.

As an example, if two enzymes (AND-logic), A and B, are catalyzing a given reaction and we want to simulate the over-expression of one of them (e.g., A), then the model assigns $P_A = 2$ (i.e., twice its wild-type probability ($P_A = 1$)). Consequently, the P-function for that reaction is represented by $P = average(P_A = 2, P_B = 1) = 1.5$. Hence, our results indicate that a systematic knockout of each enzyme shows a gradient of intermediate growth rates. This is further confirmed by **Suppl. Fig. 18**, which shows the percentage of random environments that induced intermediate growth rates between zero and the optimal growth rate for that environment. **Suppl. Fig. 19** depicts the predicted effect of TF knockouts (**Suppl. Fig. 19A**) and over-expressions (**Suppl. Fig. 19B**) to growth.

6. Layer integration under a unifying model

6.1 Integration of signal transduction, transcriptional and metabolic layers

Our approach to developing an integrative genome-scale model of *E. coli* was to divide the total functionality of the cell into modules, model each independently of the others, and integrate these sub-models together. We defined four fundamental modules (**Suppl. Fig. 20**), and independently built, parameterized, and tested a sub-model of each. A key challenge of the project was to integrate those sub-models into a unified model. To address this, we computed the growth burden due to the production and maintenance of all proteins (cost), as well as the growth advantage due to the energy uptake of the metabolic pathways in each environment (benefit).

In our cost-benefit model, the genetic cost is defined as the relative reduction in growth rate (μ) due to the production of essential proteins. We used the EBA method to predict gene expression profiles ($\hat{y}_g$) under environmental and genetic perturbations (see section 4). To measure the *cost c*, we computed the deviation between the WT (y_g^{WT}) and predicted ($\hat{y}_g$) gene expression profiles:

$$c = \frac{1}{N_G} \sum_g \left| \frac{\hat{y}_g - y_g^{\mathrm{WT}}}{y_g^{\mathrm{WT}}} \right| \qquad (25),$$

where N_G is the number of genes in *E. coli* genome. Similarly, to compute the *metabolic benefit B,* we used the metabolic sub-model described in Section 5 (equation 21). As such, the fitness function that represents the growth rate $\hat{\mu}$ is given by the difference between the benefit and the cost (10):

$$\hat{\mu} = B - c \qquad (26).$$

Suppl. Fig. 20 summarizes the information flow among the four sub-models. Environmental perturbations may modify gene expression through the signal transduction sub-model (see Section 3.2; equation 2), and may have an effect in the metabolic model by modifying directly the metabolic fluxes ($\bar{V}_{min}, \bar{V}_{max}$) according to the change of effector concentration ($\Delta\bar{n}_E$). Similarly, genetic perturbations alter the basal and regulatory coefficients ($\bar{\alpha}, \bar{\bar{\beta}}$) of the respective genes in the transcriptional model.

6.2 Model Validation

To test the utility of the integrative genome-scale model on phenotype predictions, we used a sub-set of data (295 arrays) from *Eco*MAC in which the growth rate is well characterized (i.e., *Eco*Phe; Section 1.4). We analyzed how growth rate correlates with the fitness function (equation 26) computing the *PCC* between the measured and predicted growth rates. Hence, we applied the integrated genome-scale model to predict the cost and benefit functions, and consequently, the growth rate by applying the equation 26. Then we explored the testing subset of arrays that includes the maximum number of arrays and maximized PCC. Interestingly, the growth rate of 164 arrays (from a total of 295 arrays) was predicted with high accuracy ($PCC = 0.86$, $p < 10^{-10}$, **Suppl. Fig. 21A**; right bar). To assess the predictive power of our integrated model, we generated a null-model to predict growth rates. As expected, sets of arrays with similar sizes to the previous mentioned only could achieve an accuracy of $PCC < 0.1$. Subsequently, we also analyzed how growth rate correlates with the benefit and cost functions independently. As shown in **Suppl. Fig. 21A** (middle and left bars), the predicted growth was in less agreement with the experimental value in both cases ($PCC > 0.75$, $p < 10^{-10}$; and $PCC > 0.06$, $p < 0.82$; respectively) when compared to the fitness function of equation 26, which takes both these values into account.

Then we applied the whole integrated genome-scale model to predict the cost and benefit functions. To predict gene expression, we used EBA with the TRN containing only transcriptional interactions verified experimentally. We only found 44 arrays with a maximal correlation, $PCC = 0.53$ ($p = 0.0004$), between μ and $\hat{\mu}$ (**Suppl. Fig. 21B**; right bar). Interestingly, when we added to that set the inferred interactions, growth in 81 arrays was predicted accurately with $PCC = 0.85$ ($p < 10^{-10}$) (**Suppl. Fig. 21C**; right bar).

We also studied the phenotype predictive power by focusing on different groups (**Suppl. Fig. 22**) of conditions in which: (*i*) the observed growth rate was high or low; (*ii*) the perturbations was related to environmental changes or genomic modifications; and, (*iii*) the genetic changes were gene knockouts or transcriptional rewirings.

7. Model optimization through targeted experimentation

7.1 Analysis of affected GO terms in *Eco*MAC

We explored the landscape of biological processes that could be affected by implementing all genetic perturbations contained in the training set (*Eco*MAC). For that, we used the genome ontology (GO) of EcoCyc to obtain 1,361 GO terms associated to biological processes of *E. coli*. For our purposes, we only considered GO terms related to non-specific processes. According to the Database for Annotation, Visualization and Integrated Discovery (DAVID v6.7), we only included GO terms belonged to the first five levels (686 GOs). We only identified the 80% (3,319 genes) of the *E. coli* genes related to at least one GO (**Suppl. Fig. 23A** and **23B**). We then studied the biological processes affected, according to GO and the genetic perturbations present in *Eco*MAC. For a GO process g to be affected, one of the following should happen: (a) a certain number of genes (κ) of that GO process g have been perturbed and/or (b) a certain percentage ψ of all the genes N_g^{GO} that comprise the GO process g has been perturbed:

$$\mathrm{GO}(g) = \begin{cases} \text{affected} \leftrightarrow \Phi_{\mathrm{GO}} \geq \min(\kappa,\ \psi N_g^{\mathrm{GO}}) \\ \text{non-affected} \leftrightarrow \Phi_{\mathrm{GO}} < \min(\kappa,\ \psi N_g^{\mathrm{GO}}) \end{cases} \qquad (27),$$

where Φ_{GO} is the number of genes of the GO process g that are perturbed in the dataset; N_g^{GO} is the number of genes included in $\mathrm{GO}(g)$; κ is a number of genes; and ψ is the percentage of N_g^{GO} above which we defined that the GO process is explored. Here, we set $\kappa = 3$ and $\psi = 10\%$. After testing the alteration of all GOs, we observed that only 23% (160) of all GO terms were perturbed by *Eco*MAC.

7.2 Model optimization through targeted experimentation

The integration of new experiments in the training set could improve significantly the predictive power of the *E. coli* simulator. However, selecting which experiments to perform so that the gain to the model predictive power is maximized is not a trivial task. Towards this goal, we here propose to find the set minimal set of genes that (*a*) maximizes the coverage of GO terms and (*b*) maximizes the gene expression variability that is expected in the respective arrays. For the first condition, we implemented a greedy algorithm to explore minimal sets of gene candidates to be perturbed subject to alter the maximum number of GO terms (S_2) non-altered by *Eco*MAC (77% of GO terms). Hence, optimal gene sets should minimize the scoring function:

$$S = \lambda S_1 + (1-\lambda)(S_2) \qquad (28),$$

where S_1 is the number of genes of the proposed set, S_2 is the number of GO terms non-altered by the proposed gene set according the equation 27, and λ is the weighting factor. Different sets of genes were computed by running random initializations of the greedy algorithm (**Suppl. Fig. 23C – 23D**).

Next, we explored the gene expression diversity of genetic/environmental perturbations by using the EBA method. First, we simulated all possible single gene knockouts of *E. coli* to characterize gene expression variability (V_{KO}) provided by those genetic perturbations. For that, we used EBA to predict the gene expression profile when single knockouts are implemented ($\bar{y}_{\mathrm{KO}}$), and then we calculated the relative difference between the perturbed and wild-type ($\bar{y}_{\mathrm{WT}}$) expression profiles in wild-type conditions (LB with 0.3% glucose]) given by $e_{\mathrm{KO}} = \langle(\bar{y}_{\mathrm{KO}} - \bar{y}_{\mathrm{WT}})/\bar{y}_{\mathrm{WT}}\rangle$. Here, we categorized a gene knockout as a genetic perturbation that can provide high gene expression

variability if $\frac{e_{KO}-\langle \bar{e}_{KO} \rangle}{\sigma_{\bar{e}_{KO}}} \geq 3$, where $\bar{e}_{\mathrm{KO}}$ denotes the set of relative errors provided by all gene knockouts.

Next, we calculated the gene expression diversity in other-than-WT environments for all gene knockouts. To do that, we first predicted the gene expression profiles under different environmental perturbations, by simulating the gene expression ($\bar{y}^{\mathrm{env}}$) of *E. coli* under 60 environments (see **Suppl. File 7**). Those environments were characterized by growing cells in LB media with 0.3% glucose and with the addition of high concentration of single carbon, nitrogen, oxygen, phosphate, or metal sources. We then computed the gene expression difference for each gene, *g*, between the wild-type condition and the 60 predicted environmental perturbations, as $e_g^{\mathrm{env}} = |y_g^{\mathrm{env}} - y_g^{\mathrm{WT}}|$. Analogously, we categorized all genes as potential candidates to provide high gene expression variability if $\max\left\{\frac{\eta_g-\langle \bar{\eta}_g \rangle}{\sigma_{\bar{\eta}_g}}, \frac{\vartheta_g-\langle \bar{\vartheta}_g \rangle}{\sigma_{\bar{\vartheta}_g}}\right\} \geq 3$, where η_g and ϑ_g represent the mean and standard deviation of e_g^{env} for a specific gene across the different environments respectively.

Finally, we used the criteria of maximum gene expression diversity under genetic/environmental perturbations to select the top candidates ranked to maximize the coverage of biological functions to be perturbed. This methodology has a profound effect on the number of GO terms covered and the gene expression diversity observed. As an example, by including the top 36 genetic perturbations identified by using this method, an additional 77 or 14.6% of the non-affected GO terms were perturbed (*i.e.*, the number of GO terms affected after the addition of those perturbations was 1.48-fold higher). Note that those genes were selected to maximize gene expression variability under environmental perturbations (see **Suppl. File 7**) or implementing all gene knockouts (3-fold standard deviation higher than the average in the *E. coli* genome). Interestingly, if genes are picked randomly, only the 3.3±1.1% of non-affected GO terms were perturbed (**Suppl. File 8** contains the GO terms altered by the added gene set).

7.3 Experimental measurements

Growth rates of 10 single-gene knockouts along with wild type *E. coli* MG1655 were predicted and then validated experimentally. Knockouts were chosen to ensure that they are responsive to predefined external environments. These knockouts strains were taken from Keio collection (Baba et al., 2006).

For routine culturing, cells were grown in LB medium. Knockouts were grown always in media supplemented with 50 µg/ml kanamycin unless otherwise mentioned, while wild type in media without any antibiotic. All growth experiments were done at 37^0C in M9 medium. For growth profile measurements, cells from fresh colonies were grown in 3 ml LB for 8 h, then were washed twice with glucose omitted M9 medium (Sambrook et al., 2001) to remove any trace of LB, and further dissolved in glucose omitted M9 medium. OD_{600} was measured, and depending on OD_{600}, 2 to 4 µl of cells were transferred in M9 medium to a final volume of 200 µl resulting in OD_{600} close to 0.01. Cell growth profiles were measured and recorded using Tecan infinite 200Pro microplate reader at every 10 minutes. When required, cell growth profiles were measured in M9 medium supplemented with following chemicals: *D*-glucose (0.3%), *L*-methionine (100 µg/ml), *L*-arginine (0.2%), *L*-cysteine (0.1 µM), cobalt chloride (1 µM), *L*-rhamnose monohydrate (1%),

D-ribose (0.2%), *D*-galactose (0.6%), and ferrous sulphate heptahydrate (5 mg/l). Concentrations of supplements were chosen to ensure that they were not toxic to cells.

These 10 single-gene knockout *E. coli* strains were selected from Keio collection where it was possible to record the effect of variations in external environment in form of growth rates (**Fig. 5C, Suppl. Fig. 24**). Strains with following gene deletions were used for growth rate measurements:

metN: MetN is an ATPase component of *DL*-methionine uptake system, which regulates import of *L*-methionine (Merlin et al., 2002). To see the effect of *metN* deletion on growth rate, growth rates were measured in M9 medium with or without *L*-methionine. Growth rate of MG1655 on *L*-methionine supplemented M9 medium was the highest. Growth rates of *ΔmetN* strain on M9 medium with or without *L*-methionine were similar, and were comparable to MG1655 grown on only M9 medium. These results indicate that inherent methionine biosynthesis of *E. coli* is not well sufficient to take care of methionine requirement, and MetN is solely responsible for the transport of *L*-methionine.

metL: *metL* encodes aspartate kinase II / homoserine dehydrogenase, a bifunctional enzyme that catalyzes biosynthesis of several amino acids including methionine (Theze et al., 1974). *metL* knockouts are inefficient in methionine biosynthesis, hence *metL* knockouts are expected to demonstrate retarded growth on media lacking methionine, which we observe in our growth experiments.

astE: *astE* encodes arginine succinyltransferase (AST), an enzyme involve in AST pathway of arginine degradation. AST pathway gets activated in nitrogen-limited environment. It has been reported that in media where arginine is sole source of nitrogen, *E. coli* with defects in AST pathway grows slower in the absence of arginine (Schneider et al., 1998). Our growth measurements also satisfy these facts.

cysH: *cysH* encodes PAPS reductase, which indirectly regulates cysteine synthesis. *ΔcysH* strain is not able to make cysteine (Krone et al., 1990). Our experiments demonstrated that *ΔcysH* strain grown on *L*-cysteine lacking M9 medium have slower growth rate than grown on *L*-cysteine supplemented M9 medium.

cysG: *cysG* encodes an enzyme catalyzing transformation of uroporphyrinogen III into siroheme, which indirectly governs cysteine synthesis. *ΔcysG* strain is unable to grow on cysteine lacking media, which was also observed in our experiments (Warren et al., 1990).

rhaT: *rhaT* encodes RhaT transport system that transports *L*- Rhamnose in *E. coli*. *ΔrhaT* strain is unable to transport *L*-rhamnose inside cell (Tate et al., 1992), and use this as carbon source. In our experiments, *ΔrhaT* strain grew faster on M9 medium supplemented with *D*-glucose than supplemented with *L*-rhamnose.

rbsK: *rbsK* encodes ribokinase, which catalyzes the phosphorylation of ribose to ribose 5-phosphate (Hope et al., 1986). It has been reported that *rbsK* mutants are unable to grow on media containing *D*- ribose as a sole source of carbon (Anderson and Cooper, 1969). In our experiments, *ΔrbsK* strain demonstrated much retarded growth on M9 medium containing *D*-ribose as sole source of carbon.

galK: *galK* encodes galactokinase, an enzyme involve in galactose metabolism, catalyzing phosphorylation of galactose to galactose 1-phosphate. Thus *ΔgalK* mutants are unable to metabolize galactose (Kalckar et al., 1959). In our experiments, *ΔgalK* mutants demonstrated significantly retarded growth on M9-medium containing *D*-galactose as sole source of carbon.

dgoA: *dgoA* encodes 2-oxo-3-deoxygalactonate 6-phosphate aldolase, which catalyzes degradation of *D*-galactonate, a product of *D*-galactose catabolism. Final catabolic products of *D*-galactonate enter in central metabolism (Cooper, 1978). In performed experiments, *ΔdgoA* strain demonstrated slower growth rate on M9 medium containing *D*-galactose as sole source of carbon, because due to *dgoA* deletion one of several pathways of *D*-galactose degradation was blocked, leading to less degradation of *D*-galactose

mntH: *mntH* encodes a divalent metal ion transporter, which transports Mg^{2+}, and Fe^{2+} (Makui et al., 2000). In our experiments, MG1655 and *ΔmntH* strains demonstrated faster growth rates on $FeSO_4$ supplemented M9 medium, compared to medium lacking $FeSO_4$.

Note that all genes selected are metabolic enzymes and they were found in iJO1366 metabolic model. Consequently, those gene knockouts did not induce apparent effects in the gene expression profiles predicted by EBA under WT media. However, gene expression patterns of those knockout strains simulated in supplemental media showed significant variations with respect to the WT, because of media supplements introduced changes in gene expression directly in some TFs (*creB, uhpA, meJ, argP, metR, nikR, rcnR, rhaR, rhaS, rbsR, rpiR, galR, galS, glpR, gatR_1, iscR,* and *nsrR*) through the signal transduction sub-model.

OD_{595} nm as a measure of the cell growth was represented on a logarithmic scale to highlight strictly exponential growth phase, which was selected for calculation of growth rate by using 3 replicates.

7.4 Comparison between experimental and predicted cell growth values

We applied our integrated model to predict the growth rate of 28 different phenotypes. Specifically, we predicted both WT and the 10 single-knockout strains in (*a*) M9 salt medium supplemented with 0.3% glucose (10+1 strains) and (*b*) in the previous medium (M9 and 0.3% glucose) with the addition of a single compensating chemical as described in section 7.3.

To simulate the growth rate for the previous 28 phenotypes, we ran the integrated genome-scale model as follows: (*i*) modification of the equations (15-16) from the EBA to predict the gene expression profiles under the gene knockouts and environments defined by the addition of carbon sources and the eight chemicals used; (*ii*) we applied TRAME to compute the metabolic flux bounds according to the genetic and environmental perturbations; and then, (*iii*) we computed the benefit and cost (see Section 6) to predict the growth rate.

Having considered these above-described model predictions for the WT *E. coli* strain and 29 mutants, we measured the growth rates for the phenotypes mentioned previously (**Suppl. Fig. 24**). Interestingly, the model accounts for previously observed gene essentiality with 79% accuracy ($p < 10^{-4}$; **Fig. 5D**) across the 28 phenotypes experimentally explored. The model predictions showed discrepancies with measured growth rates ($\hat{\mu}$) non-significant ($p > 10^{-3}$; $\hat{\mu} \in [\mu - 3\sigma_{\mu}, \mu + 3\sigma_{\mu}]$) in less than 43% of the phenotypes with a low growth rate measured (28 measurements). Interestingly, the model predictions showed discrepancies with measured growth rates non-significant in less than 75% and 58% of the phenotypes related to genetic perturbations (20 measurements) and gene knockouts (10 measurements) respectively. Only the 53% of

phenotypes (17 measurements), in which environmental perturbations play a role, were predicted with statistical significance (**Fig. 5D**).

We also examined the gene expression profiles predicted by EBA for these 30 phenotypes. A total of 149 genes were significantly altered ($p < 10^{-3}$) in those conditions (**Suppl. File 8**). Next, we performed a functional enrichment of those genes and different GO terms related were identified as pathways potentially altered ($p < 10^{-3}$) in the signal transduction systems and TRN (e.g., response to external stimulus), or metabolism (e.g., primary metabolic process, carbohydrate and amino acid metabolic and catabolic process) of *E. coli.*

References

Anderson A, Cooper RA (1969) The significance of ribokinase for ribose utilization by *Escherichia coli*. *Biochim Biophys Acta* **177:** 163-165

Baba T, Ara T, Hasegawa M, Takai Y, Okumura Y, Baba M, Datsenko KA, Tomita M, Wanner BL, Mori H (2006) Construction of *Escherichia coli* K-12 in-frame, single-gene knockout mutants: the Keio collection. *Mol Syst Biol* **2:** 2006 0008

Carrera J, Elena SF, Jaramillo A (2012) Computational design of genomic transcriptional networks with adaptation to varying environments. *Proc Natl Acad Sci USA* **109:** 15277-15282

Carrera J, Rodrigo G, Jaramillo A, Elena SF (2009) Reverse-engineering the *Arabidopsis thaliana* transcriptional network under changing environmental conditions. *Genome Biol* **10:** R96

Cooper RA (1978) The utilisation of D-galactonate and D-2-oxo-3-deoxygalactonate by *Escherichia coli* K-12. Biochemical and genetical studies. *Arch Microbiol* **118:** 199-206

Faith JJ, Driscoll ME, Fusaro VA, Cosgrove EJ, Hayete B, Juhn FS, Schneider SJ, Gardner TS (2008) Many Microbe Microarrays Database: uniformly normalized Affymetrix compendia with structured experimental metadata. *Nucleic Acids Res* **36:** D866-870

Faith JJ, Hayete B, Thaden JT, Mogno I, Wierzbowski J, Cottarel G, Kasif S, Collins JJ, Gardner TS (2007) Large-scale mapping and validation of *Escherichia coli* transcriptional regulation from a compendium of expression profiles. *PLoS Biol* **5:** e8

Greenfield A, Madar A, Ostrer H, Bonneau R (2010) DREAM4: Combining genetic and dynamic information to identify biological networks and dynamical models. *PLoS One* **5:** e13397

Gudmundsson S, Thiele I (2010) Computationally efficient flux variability analysis. *BMC Bioinformatics* **11:** 489

Haury AC, Mordelet F, Vera-Licona P, Vert JP (2012) TIGRESS: Trustful Inference of Gene REgulation using Stability Selection. *BMC Syst Biol* **6:** 145

Hope JN, Bell AW, Hermodson MA, Groarke JM (1986) Ribokinase from *Escherichia coli* K12. Nucleotide sequence and overexpression of the *rbsK* gene and purification of ribokinase. *J Biol Chem* **261:** 7663-7668

Huynh-Thu VA, Irrthum A, Wehenkel L, Geurts P (2010) Inferring regulatory networks from expression data using tree-based methods. *PLoS One* **5**

Kalckar HM, Kurahashi K, Jordan E (1959) Hereditary Defects in Galactose Metabolism in *Escherichia coli* Mutants, I. Determination of Enzyme Activities. *Proc Natl Acad Sci USA* **45:** 1776-1786

Keseler IM, Mackie A, Peralta-Gil M, Santos-Zavaleta A, Gama-Castro S, Bonavides-Martinez C, Fulcher C, Huerta AM, Kothari A, Krummenacker M, Latendresse M, Muniz-Rascado L, Ong Q, Paley S, Schroder I, Shearer AG, Subhraveti P, Travers M, Weerasinghe D, Weiss V *et al* (2013) EcoCyc: fusing model organism databases with systems biology. *Nucleic Acids Res* **41:** D605-612

Krone FA, Westphal G, Meyer HE, Schwenn JD (1990) PAPS-reductase of *Escherichia coli*. Correlating the N-terminal amino acid sequence with the DNA of gene *cys H*. *FEBS Lett* **260:** 6-9

Kuffner R, Petri T, Tavakkolkhah P, Windhager L, Zimmer R (2012) Inferring gene regulatory networks by ANOVA. *Bioinformatics* **28:** 1376-1382

Mahadevan R, Schilling CH (2003) The effects of alternate optimal solutions in constraint-based genome-scale metabolic models. *Metab Eng* **5:** 264-276

Makui H, Roig E, Cole ST, Helmann JD, Gros P, Cellier MF (2000) Identification of the *Escherichia coli* K-12 Nramp orthologue (MntH) as a selective divalent metal ion transporter. *Mol Microbiol* **35:** 1065-1078

Marbach D, Costello JC, Kuffner R, Vega NM, Prill RJ, Camacho DM, Allison KR, Kellis M, Collins JJ, Stolovitzky G (2012) Wisdom of crowds for robust gene network inference. *Nat Methods* **9:** 796-804

Merlin C, Gardiner G, Durand S, Masters M (2002) The *Escherichia coli metD* locus encodes an ABC transporter which includes Abc (MetN), YaeE (MetI), and YaeC (MetQ). *J Bacteriol* **184:** 5513-5517

Orth JD, Conrad TM, Na J, Lerman JA, Nam H, Feist AM, Palsson BO (2011) A comprehensive genome-scale reconstruction of *Escherichia coli* metabolism--2011. *Mol Syst Biol* **7:** 535

Salgado H, Peralta-Gil M, Gama-Castro S, Santos-Zavaleta A, Muniz-Rascado L, Garcia-Sotelo JS, Weiss V, Solano-Lira H, Martinez-Flores I, Medina-Rivera A, Salgado-Osorio G, Alquicira-Hernandez S, Alquicira-Hernandez K, Lopez-Fuentes A, Porron-Sotelo L, Huerta AM, Bonavides-Martinez C, Balderas-Martinez YI, Pannier L, Olvera M *et al* (2013) RegulonDB v8.0: omics data sets, evolutionary conservation, regulatory phrases, cross-validated gold standards and more. *Nucleic Acids Res* **41:** D203-213

Sambrook J, Russell DW (2001) Molecular Cloning: A laboratory manual. 3rd edition. New York: Cold Spring Harbor Laboratory Press.

Savageau MA (1998a) Demand theory of gene regulation. I. Quantitative development of the theory. *Genetics* **149:** 1665-1676

Savageau MA (1998b) Demand theory of gene regulation. II. Quantitative application to the lactose and maltose operons of *Escherichia coli*. *Genetics* **149:** 1677-1691

Schneider BL, Kiupakis AK, Reitzer LJ (1998) Arginine catabolism and the arginine succinyltransferase pathway in *Escherichia coli*. *J Bacteriol* **180:** 4278-4286

Segre D, Vitkup D, Church GM (2002) Analysis of optimality in natural and perturbed metabolic networks. *Proc Natl Acad Sci USA* **99:** 15112-15117

Tate CG, Muiry JA, Henderson PJ (1992) Mapping, cloning, expression, and sequencing of the *rhaT* gene, which encodes a novel L-rhamnose-H+ transport protein *in Salmonella typhimurium* and *Escherichia coli*. *J Biol Chem* **267:** 6923-6932

Theze J, Margarita D, Cohen GN, Borne F, Patte JC (1974) Mapping of the structural genes of the three aspartokinases and of the two homoserine dehydrogenases of *Escherichia coli* K-12. *J Bacteriol* **117:** 133-143

Warren MJ, Roessner CA, Santander PJ, Scott AI (1990) The *Escherichia coli cysG* gene encodes S-adenosylmethionine-dependent uroporphyrinogen III methylase. *Biochem J* **265:** 725-729

Yilmaz A, Mejia-Guerra MK, Kurz K, Liang X, Welch L, Grotewold E (2011) AGRIS: the Arabidopsis Gene Regulatory Information Server, an update. *Nucleic Acids Res* **39:** D1118-1122
