# Supplementary material for: An integrative, multi-scale, genome-wide model reveals the phenotypic landscape of Escherichia coli
Source: Mol Syst Biol. 2014 Jul 1;10(7):735. doi: 10.15252/msb.20145108 (PMC4299492; doi:10.15252/msb.20145108)
Supplement: Supplementary file 13 — Supplementary Dataset S10 [file msb0010-0735-sd13.zip › Models and code/README.pdf]

The integrative, multi-scale genome-scale model reveals the phenotypic landscape of *Escherichia coli*

Javier Carrera,[1,4] Raissa Estrela,[2] Jing Luo,[1] Navneet Rai,[1] Athanasios Tsoukalas,[1,3] Ilias Tagkopoulos,[1,3*]

This integrative model of *E. coli* is licensed under a free Creative Commons Attribution-Noncommercial 3.0. License (http://creativecommons.org/licenses/by-nc/3.0). If you use this software for your research, please provide the appropriate reference to this work.

Manual to run the all sub-models of the integrative *E. coli* model

Overview. This integrative model of *E. coli* is MatLab program aimed at the simulation of the well-characterized cellular processes of *E. coli* under genetic and environmental perturbations. This software is based on the integration of four genome-scale models (EBA, TRAME, FVA and FBA) to predict phenotype of *E. coli*. It uses a registry of models to represent gene expression, transcriptional regulation, signal transduction and metabolic processes. The software writes the predicted phenotype.

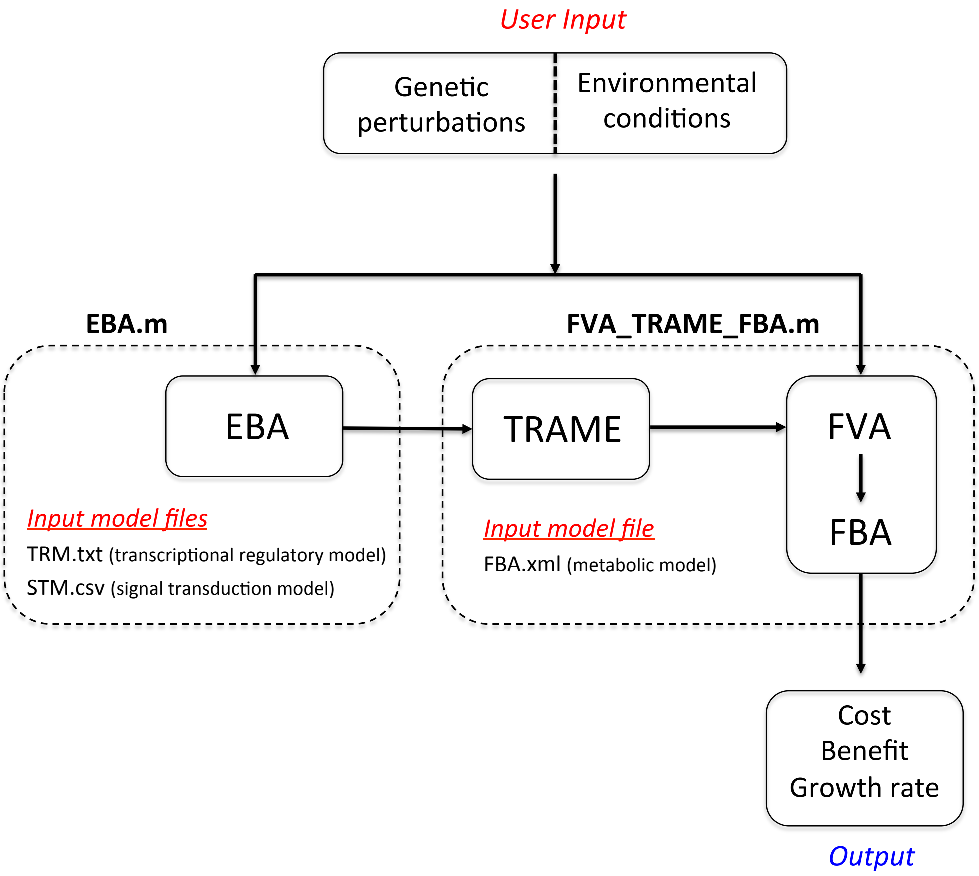

Pipeline of the different sub-models implemented in MatLab code.

Input files. The integrative model of *E. coli* reads three genome-scale models provided in the directory models/

i) TRM_1.txt (only experimental interactions from RegulonDB) and TRM_2.txt (experimental and inferred interactions) contain the parameters (α, β; Suppl. Methods, section 4.4) specified in the transcriptional model as a matrix with dimensions, (number of genes) x (number of transcription factors). The order of genes is provided in the file numbering_assembly-tfs-enzymes-genes.txt (Supplementary file 1).
ii) STS_1.txt and STS_2.txt are files containing the parameters (χ, τ, respectively; Suppl. Methods, section 3.1) from the signal transduction systems.
iii) FBA.txt is the metabolic model from Orth, J.D., et al. (2011) *Mol Syst Biol* **7**:535.

Additionally, models/ should contain the gene expression matrix of *Eco*MAC provided in the Supplementary file 1.

Pre-installation requirements in MatLab to run the integrated model:

1. Download (http://opencobra.sourceforge.net/openCOBRA/Welcome.html) and setup CobraToolbox.
2. Download (https://notendur.hi.is/ithiele/software/fastfva.html) and setup fastFVA.

Execution of the program for simulation

❖ Execute EBA as follows:

```
> GeneExpression = EBA (x, y, w, z, t, RNA, PROTEIN);
```

1) ***x*** is the parameter (ϕ) that defines gene expression bounds (C_{min} and C_{max});
2) ***y*** is a flag defining the topology of the transcriptional model, ***y = 0*** (experimental interactions from RegulonDB, TRM_1.txt), and ***y = 1*** (interactions experimentally verified and inferred, TRM_2.txt);
3) ***w*** is a flag that defines the medium, ***w = 0*** (LB) and ***w = 1*** (M9);
4) ***z*** is a two column-vector that defines the *N*-genetic perturbations where the first column-vector contains the gene perturbed ($z(i,1) \in [1,\ 4{,}189]$) and the second vector is $z(i,2) = -1$ (gene knockout), $z(i,2) = 0$ (overexpression), a number between 1 and 4,189 that defines the promoter controlling the expression of a given TF ($z(i,2) \in [1,\ 4{,}189]$, i.e., TF rewiring);
5) ***t*** is a matrix that defines the transcription factors (TFs) interacting with the environmental factors (EFs) related to the supplemental nutrients specified for each environment. The dimensions are *N* (number of perturbations simulated) x (number of TFs interacting with the EFs by the signal transduction systems specified in the STS.txt), then $t(i,j) \in [1,\ 328]$;

6) ***RNA*** is a matrix with same the dimensions than ***t*** that represents the parameters χ of the signal transduction systems;
7) ***PROTEIN*** is a matrix with same the dimensions than ***t*** that represents the parameters τ of the signal transduction systems.

❖ Execute FVA, TRAME and FBA:

```
> [cost, benefit, growth] = FVA_TRAME_FBA (z, EF);
```

1) z contains the same information than the previous one defined;
2) ***EF*** is a matrix that defines the EFs related to the supplemental nutrients specified for each environment. The dimensions are N (number of perturbations simulated) x (number of EFs or metabolic uptakes specified in metabolic model), then $EF(i,j) \in [9,\ 332]$;.

Output files. The model outputs different files with the predicted phenotype:

i) Gene expression: GeneExpression.txt (The order of genes is provided in the file numbering_assembly-tfs-enzymes-genes.txt (Supplementary file 1)).
ii) Growth rate: GrowthRate.txt provides the values for the predicted cost, benefit and growth rate (Suppl. Methods, section 6.1).

Example. We show different simulations presented in the validation by targeted experimentation:

Simulation	**Gene perturbed**	Gene ID	Type of genetic perturbation	**Supplemental nutrients**	EF ID (i.e., metabolic uptake)	TF ID affected by the EF
#1	**metN**	417	-1	**L-methionine**	229	278
#2	**metL**	1540	-1	**L-methionine**	229	278
#3	**cysG**	1324	-1	**CoCl2**	86	242, 149
#4	**astE**	873	-1	**arginine**	66	227, 203
#5	**rhaT**	1526	-1	**L-rhamnose**	282	275, 274
#6	**cysH**	1179	-1	**L-cysteine**	98	267
#7	**rbsK**	1472	-1	**D-ribose**	281	264, 290
#8	**galK**	590	-1	**D-galactose**	152	239, 147, 198, 152
#9	**dgoA**	1680	-1	**D-galactose**	152	239, 147, 198, 152
#10	**mntH**	1063	-1	**FeSO4**	128	50, 173, 214
#11	**cysG**	1324	-1	**D-cysteine**	98	267
#12	**strain WT**	-	-	**-**	-	-
#13	**strain WT**	-	-	**L-methionine**	229	278

#14	**strain WT**	-	-	**CoCl2**	86	242, 149
#15	**strain WT**	-	-	**L-arginine**	66	227, 203
#16	**strain WT**	-	-	**L-rhamnose**	282	275, 274
#17	**strain WT**	-	-	**L-cysteine**	98	267
#18	**strain WT**	-	-	**D-ribose**	281	264, 290
#19	**strain WT**	-	-	**D-galactose**	152	239, 147, 198, 152
#20	**strain WT**	-	-	**FeSO4**	128	50, 173, 214

Note that Genes and TFs are represented by the IDs from the list of *E. coli* genes provided in the **Supplementary file 1**. EFs are represented by the metabolic uptakes associated to the metabolic model. To find the TFs affected by a given EF, we used the information about the STSs in the **Supplementary file 5**, or STS_i.csv files.

```
> x = 0.9;        % Φ = 0.9

> y = 0;          % Experimental interactions

> w = 0;          % LB medium

> z = [417 -1; 1540 -1; 1324 -1; 873 -1; 1526 -1; 1179
-1; 1472 -1; 590 -1; 1680 -1; 1063 -1; 1324 -1; 0 -
1; 0 -1; 0 -1; 0 -1; 0 -1; 0 -1; 0 -
1; 0 -1; 0 -1;]; % Genetic perturbations

> t = [278 0 0 0; 278 0 0 0; 242 149 0 0; 227 203 0
0; 275 274 0 0; 267 0 0 0; 264 290 0 0; 239 147
198 152; 239 147 198 152; 50 173 214 0; 267 0 0 0;
     0 0 0 0; 278 0 0 0; 242 149 0 0; 227 203 0 0;
     275 274 0 0; 267 0 0 0; 264 290 0 0; 239 147
198 152; 50 173 214 0;]; % Environmental perturbations (TFs
affected)

> RNA = [0 0 0 0; 0 0 0 0; 0 1 0 0; -1 0 0 0; 1 1 0 0;
     -1 0 0 0; 1 0 0 0; 0 0 1 1; 0 0 1 1; -1 -1 0 0;
     -1 0 0 0; 0 0 0 0; 0 0 0 0; 0 1 0 0; -1 0 0 0; 1
     1 0 0; -1 0 0 0; 1 0 0 0; 0 0 1 1; -1 -1 0 0;];

> PROTEIN = [1 0 0 0; 1 0 0 0; 1 -1 0 0; 1 1 0 0; 1 1
0 0; 1 0 0 0; -1 0 0 0; -1 0 -1 -1; -1 0 -1 -1; 1 1
-1 0; 1 0 0 0; 0 0 0 0; 1 0 0 0; 1 -1 0 0; 1 1 0 0;
     1 1 0 0; 1 0 0 0; -1 0 0 0; -1 0 -1 -1; 1 1 -1
0;];

> GeneExpression = EBA (x, y, w, z, t, RNA, PROTEIN);

> EF = [229 229 86 66 282 98 281 152 152 128
98 0 229 86 66 282 98 281 152 128]; %
Environmental perturbations (EFs)
```

```
>[Cost, Benefit, GrowthRate] = FVA_TRAME_FBA (z, EF);
```
