# Supplementary material for: An integrative, multi-scale, genome-wide model reveals the phenotypic landscape of Escherichia coli
Source: Mol Syst Biol. 2014 Jul 1;10(7):735. doi: 10.15252/msb.20145108 (PMC4299492; doi:10.15252/msb.20145108)
Supplement: Supplementary file 15 — Supplementary File Legends [file msb0010-0735-sd15.pdf]

Supplementary File legends

Supplementary file S1: Compressed files of the *Eco*MAC transcriptional compendium. Text files contain normalized gene expression values. The 4,189 rows and 2,189 columns correspond to genes and arrays, respectively.

Supplementary file S2: Description of the 2,189 arrays included in the EcoMAC and EcoPhe compendia.

Supplementary file S3: Transcriptional regulatory interactions that have been verified experimentally.

Supplementary file S4: Inferred transcriptional interactions based on *Eco*MAC expression data.

Supplementary file S5: The EcoST Signal transduction compendium.

Supplementary file S6: Description of the metabolic sources and experiments used for environmental prediction (Section 5).

Supplementary file S7: Ranked list of genes that maximize (i) the GO terms coverage, (ii) gene expression diversity in gene knockouts and (iii) the gene expression diversity under environmental conditions (Section 7).

Supplementary file S8: List of genes and biological processes that were significantly altered after inclusion of the new phenotypes that were experimentally measured.

Supplementary file S9: Growth rate measurements for the 28 phenotypes (section 7.4).

Supplementary file S10: MatLab code for the integrative model containing the two novel methods, EBA and TRAME, and Cytoscape files for the network showed in **Fig. 3** and **5**.

Supplementary file S11: Correlations between measured and predicted growth rates by using M, ME models and the integrative model, and gene essentiality evaluated by the integrative model.
